# Integrated analysis to study the interplay between post-translational modifications (PTM) in hepatitis C virus proteins and hepatocellular carcinoma (HCC) development

**DOI:** 10.1038/s41598-022-19854-6

**Published:** 2022-09-19

**Authors:** Aqsa Ikram, Bisma Rauff, Badr Alzahrani, Faryal Mehwish Awan, Ayesha Obaid, Anam Naz, Salik Javed Kakar, Hussnain Ahmed Janjua

**Affiliations:** 1 Institute of Molecular Biology and Biotechnology (IMBB), University of Lahore (UOL), Lahore, Pakistan; 2 Department of Biomedical Engineering, UET Lahore, Narowal campus, Narowal, Pakistan; 3 Department of Clinical Laboratory Sciences, Jouf University, Sakaka, Saudi Arabia; 4 Department of Medical Lab Technology, University of Haripur (UOH), Haripur, Pakistan; 5 Atta Ur Rahman School of Applied Biosciences (ASAB), National University of Sciences and Technology (NUST), Islamabad, Pakistan

**Keywords:** Computational biology and bioinformatics, Structural biology, Systems biology, Biomarkers

## Abstract

Many PTMs dysregulation is known to be the major cause of many cancers including HCV induced HCC. PTMs of hepatitis C virus (HCV) regions NS3/4A, NS5A and NS5B are crucial for proper protein functions and replication that directly affect the generation of infectious virus particles and completion of its life cycle. In this study, we have performed comprehensive analysis of PTMs within HCV non-structural proteins (NS3/4A, NS5A and NS5B) through bioinformatics analysis to examine post-translational crosstalk between phosphorylation, palmitoylation, methylation, acetylation and ubiquitination sites in selected viral proteins. Our analysis has revealed many highly putative PTMs sites that are also conserved among major genotypes conferring the importance of these sites. We have also analysed viral 3D structures in their modified and unmodified forms to address extent and signatures of structural changes upon PTM. This study provides evidence that PTMs induce significant conformational changes and make viral proteins more stable. To find the potential role of PTMs in HCV induced HCC, docking analysis between selected viral proteins and p38-MAPK has been performed which also confirms their strong association with HCV induced HCC. The major findings proposed that PTMs at specific sites of HCV viral proteins could dysregulate specific pathways that cause the development of HCC.

## Introduction

Hepatitis C virus (HCV) is a positive sense RNA virus. It belongs to the family *Flaviviridae*^1^. It develops chronic infection in humans that leads to hepatic failure^2^. So far, the only HCV treatment is combination therapy of pegylated interferon and ribavirin (PEG/RBV), however, direct-acting antivirals (DAAs) has initiated the era of well-tolerated medications^3^. Though these treatments are effective but still encounter certain limitations including drug resistance, mutations and side effects^4^. Hence, for the development of an efficient antiviral treatment, comprehensive knowledge of viral pathogenesis is essential.

**Figure 1:**
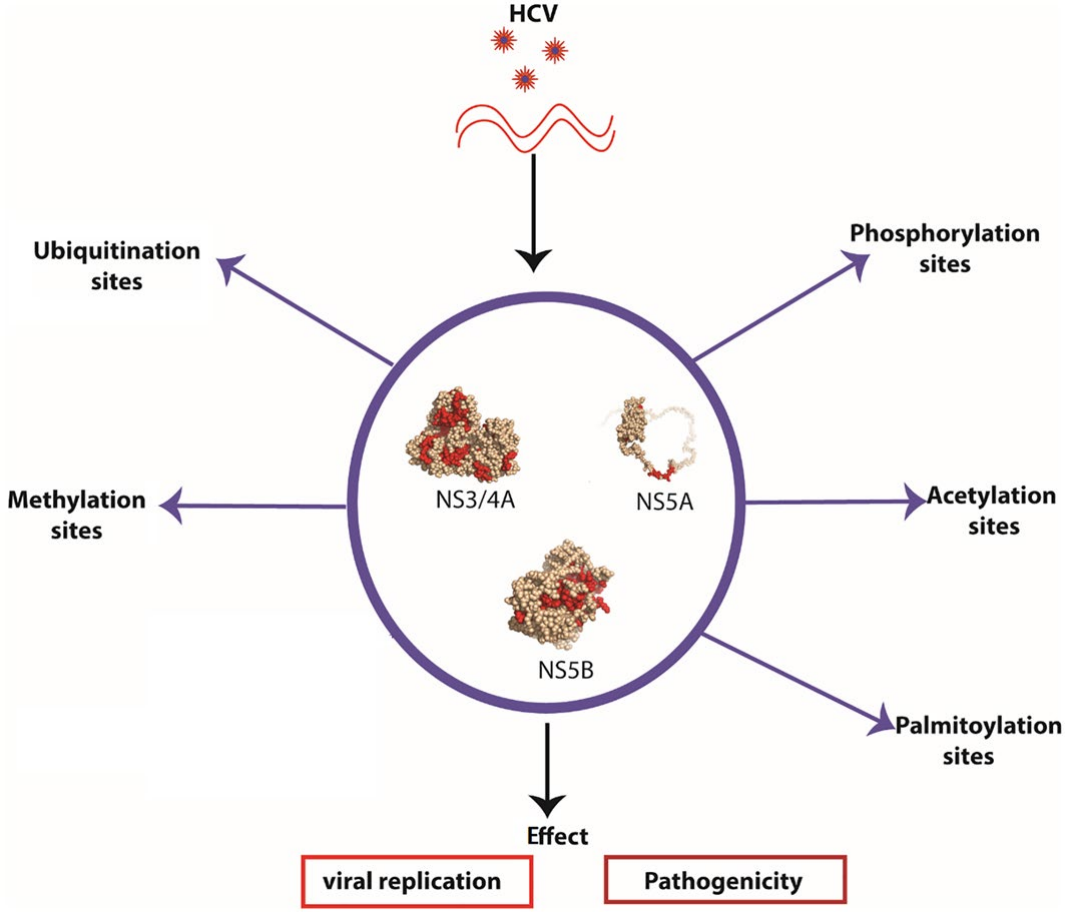
Schematic illustration of potential PTMs within HCV NS3/4A, NS5A, NS5B. The PTMs significantly affect the viral replication and pathogenesis.

Post-translational modifications (PTMs) are covalent interactions that occur either as a result of addition of modifying groups or by proteolytic cleavage that ultimately alters the main properties of proteins. Addition of simple chemical groups include acetyl, hydroxyl, methyl, or phosphate groups, however, addition of more complex groups include AMP, ADP-ribose, lipids, sugars and small polypeptides like ubiquitins^5^. PTMs are known to provide essential insights of various cellular functions^6^, including protein–protein interactions, their localization and turnover. In addition, PTMs are substantial approaches that can be used by many pathogens, both by viruses and bacteria, to control host factors that are important for their infection^7^. Viruses rely on the protein synthesis machinery of host cells to support their replication, and not surprisingly, many viral proteins are subjected to PTMs. PTMs of viral and cellular proteins have gained increasingly attention as modifying enzymes regulate virtually every step of the viral replication cycle. Furthermore, it is also recognised that PTMs dysregulation could lead to serious diseases including the development of cancers^8^. Among the various PTMs, phosphorylation is one of the most widely studied PTM and its alteration has been observed in cancers including HCC, which is a multi-event process and is caused majorly by HCV and HBV. In HCV induced HCC, PTMs, specifically, phosphorylation is known to play a key role towards liver pathogenesis^9–11^. These alterations mostly occur in important proteins involved in cellular pathways. In addition, few HCV proteins including HCV NS3/4A, NS5A and NS5B are also known to play a critical role towards HCV induced HCC^12^. Many PTMs have been reported against HCV viral proteins in various studies and include acetylation, methylation, phosphorylation, palmitoylation, and ubiquitination^7^. These modifications confirmed many protein functions by regulating protein activity, protein nucleic acid interaction, protein–protein interactions, subcellular localization and replication^7^. However, there are very few studies conducted in relevant to the PTMs sites in HCV viral proteins and their connection with HCV mediated HCC. Therefore, in this study we have performed comprehensive analysis of PTMs within HCV non-structural proteins (NS3/4A, NS5A and NS5B) that are mainly involved in HCV replication and interact with key cellular proteins (Fig. 1). Our analysis showed that there are several putative sites in the selected viral proteins that have high potential of PTMs site. Also, many of these sites were conserved among major genotypes (1–7), conferring the importance of these sites. The docking analysis of selected viral proteins with MAPK-p38 also revealed their strong interaction at the predicted PTMs sites representing their potential role in HCV induced HCC. Therefore, in this study it was revealed that PTM sites and PTM pathways can be possible targets for antiviral drug development against HCV induced HCC^13^. In this instance, identification and detailed understanding of HCV-mediated PTMs within NS3/4A, NS5A and NS5B will provide valuable information for future antiviral drug development. Indeed, careful selection and inhibition of these sites may develop an optimistic strategy to counter these deceptive invaders.

## Results

### Analysis of phosphorylation sites

**Figure 2:**
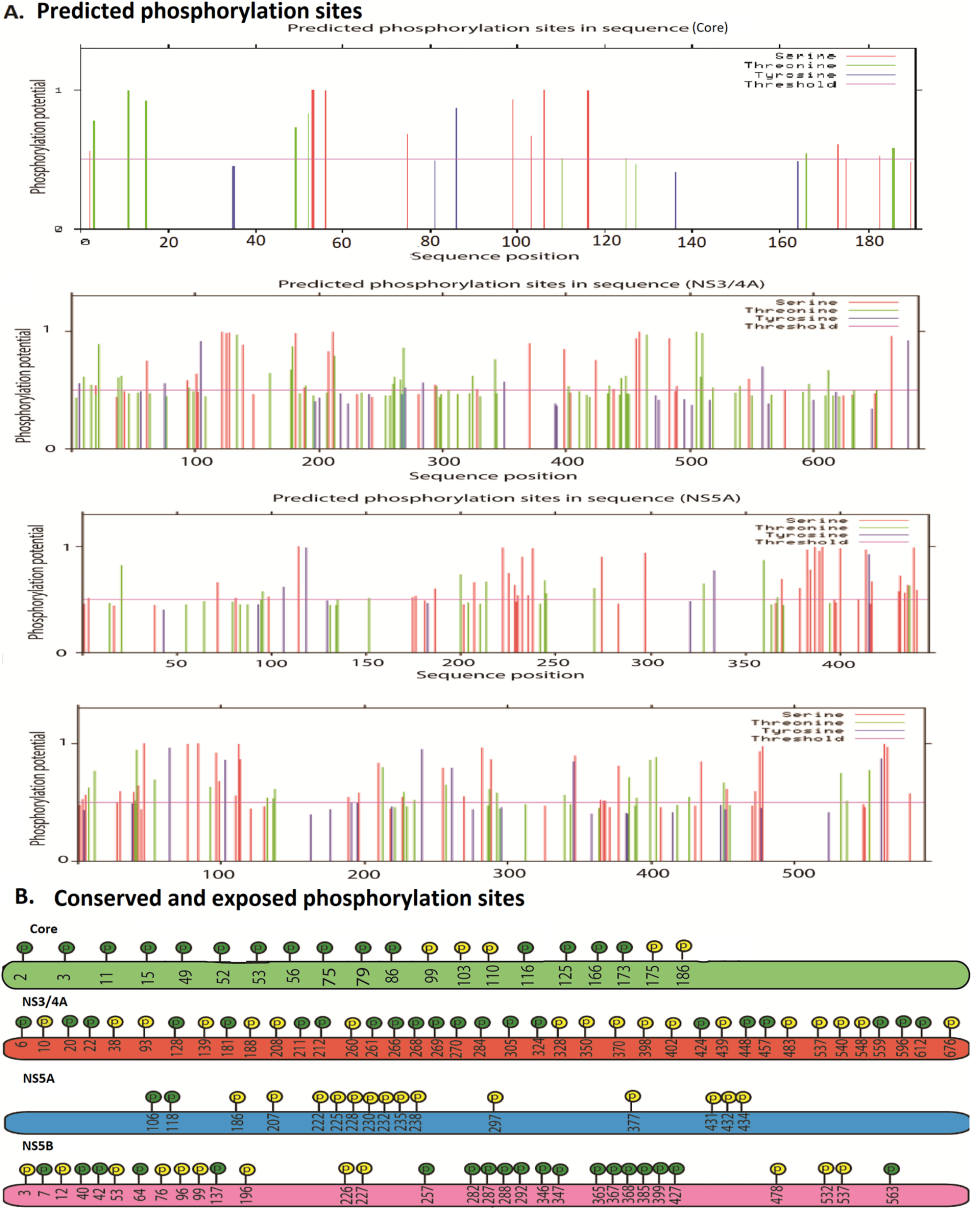
Predicted and conserved phosphorylation sites (**A**) A graphical presentation of all predicted phosphorylation sites within HCV Core, NS3/4A, NS5A and NS5B. (**B**) Conserved phosphorylation sites: yellow color phosphorylations sites are also exposed in viral proteins.

In core nineteen sites are predicted to be high phosphorylated sites which are also conserved among the seven genotypes (Table S1) (Table S1 highlighted in pink color). Sixty-eight sites within NS3/4A region reveals high potential for phosphorylation (Table S2). Among these sites thirty-nine are found to be conserved in genotypes (1–7) (Table S2 highlighted in yellow color; Fig. 2). In NS5A, fifty-one sites are predicted to be potential phosphorylated sites, while only sixteen sites are conserved among major genotypes (Table S3 highlighted in orange color; Fig. 2). On analysing NS5B region, it has been revealed that 87 putative phosphorylation sites are found. Among them, 31 are conserved among all genotypes, (Table S4 highlighted in green color, Fig. 2). To determine the surface accessibility of potential phosphorylation sites is of great importance, as the residues that are hidden or buried in the core of protein would have less chances to be exposed to any kinase. To validate the surface availability of predicted phosphorylated sites Netsurf has been used. In our analysis, it is observed that in core Ser2, Thr3, Thr11, Thr49, Thr52, Ser52, Ser56, Ser75, Tyr86, Thr110, Ser116, Thr125, Thr166, Thr166, Ser173, Ser175, Thr186 (Table S1) residues are conserved and exposed while in NS3/4A region, 18 conserved phosphorylated sites (Thr10, Thr38, Ser93, Ser139, Ser188, Ser208, Thr260, Thr328, Tyr350, Ser370, Ser398, Thr402, Ser439, Ser483, Thr537, Thr540, Ser548, Tyr676) are also exposed (Table S1 Fig. 2). Within NS5A protein, 15 conserved putative phosphorylation sites are observed (Ser186, Ser207, Ser222, Ser225, Ser228, Ser230, Ser232, Ser235, Ser238, Ser297, Thr377, Ser431, Ser432, Ser432) (Table S3, Fig. 2). For NS5B region, 12 conserved phosphorylated sites have been revealed (Ser3, Thr12, Thr53, Ser76, Ser96, Ser 99, Ser196, Ser226, Thr227, Ser478, Thr532, Thr537) (Table S4, Fig. 2). These conserved sites could be available for phosphorylation for all viral genotypes.

### Analysis of acetylation and palmitoylation sites

**Figure 3:**
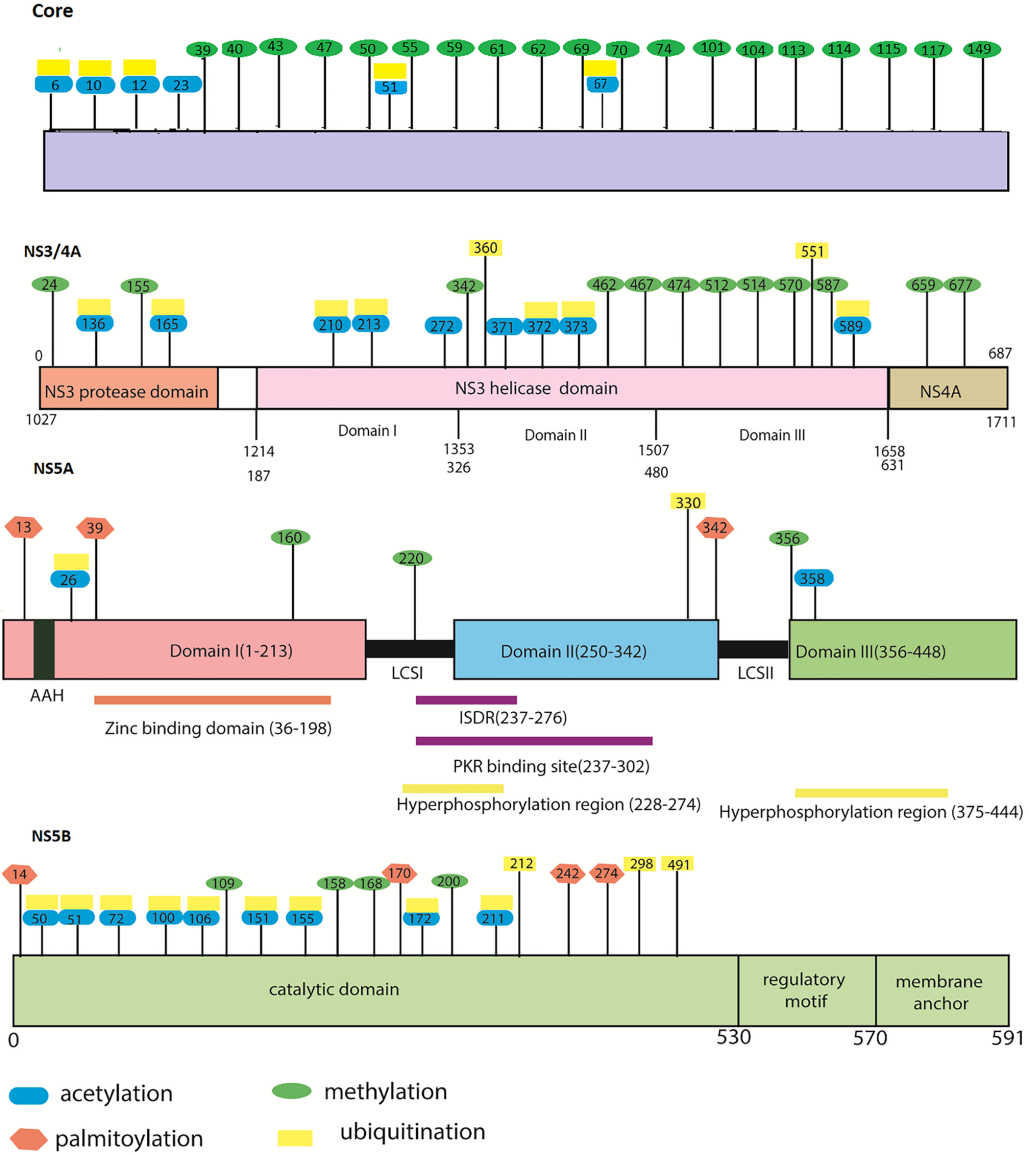
Schematic representation of predicted conserved acetylation, methylation, palmitoylation and ubiquitination sites within HCV NS3/4A, NS5A and NS5B. Blue oval shape represents acetylation sites, pink hexagon represents palmitoylation sites, green sphere represents methylation sites and yellow rectangular shapes are ubiquitination sites.

Another common post-translational modification of proteins is the acetylation of lysine residue. In our analysis, CSS-PALM 3.0 server predicts many possible acetylation sites within selected viral proteins (Core, NS3/4A, NS5A and NS5B). Six conserved acetylation sites has been observed in Core protein (6, 10, 12, 23, 51, 67) Total fourteen sites in NS3/4A region are found to be putative acetylation sites. Among them, nine sites are conserved in major genotypes (136, 165, 210, 213, 272, 371, 372, 373, 589) (Fig. 3; blue oval shape, Table S5; highlighted in blue colour). In NS5A region, seven sites are predicted to be acetylated sites, while only two (26, 285) are found to be conserved in genotypes (Fig. 3; blue oval shape, Table S5; highlighted in blue colour). Twenty-two, acetylated sites are observed in NS5B and nine (50, 51, 72, 100, 106, 151, 155, 172, 211) (Fig. 3; blue oval shape, Table S5; highlighted in blue colour) are conserved within the genotypes, conferring the importance of these sites.

Palmitoylation is a unique reversible cysteine thioacylation adjustment that involves covalent attachment of fatty acid, primarily palmitic acid, to the protein molecule^14^. In Core and NS3/4A, no palmitoylation sites are observed. In NS5A, only three (13 Cys, 39 Cys, 342 Cys) and in NS5B four putative sites (Cys14, 170 Cys, 242 Cys, 274 Cys) are observed (Table S6; highlighted in pink colour, Fig. 3; pink hexagon). Among them, all the sites in NS5A are conserved, while only one site (Cys170) is found to be conserved in NS5B (Table S6, Fig. 3; pink hexagon).

### Analysis of methylation sites

PMes server predict 19 methylation sites (39, 40, 43, 47, 50, 55, 59, 61, 62, 69, 70, 72, 74, 101, 104, 113, 114, 115, 117, 149 ) in core protein which are also conserved among all genotypes. This prediction also revealed 12 methylation sites in NS3/4A, while 10 predicted sites at position 24, 155, 462, 474, 512, 514, 570, 587, 659, 677 are conserved among all genotypes (Table S7; highlighted in purple colour, Fig. 3; green spheres). In NS5A, total seven sites are found to have high potential for methylation, while, R residue at position 220 and 356 are conserved among genotypes (Table S7; highlighted in purple colour, Fig. 3; green spheres). In NS5B, six methylation sites are predicted and R residue at positions 109, 158, 168, and 200 are also found to be conserved (Table S7; highlighted in purple colour, Fig. 3; green spheres).

### Analysis of ubiquitination sites

In Core only five sites (6, 10, 12, 51, 67) are possible ubiquitination sites which are also conserved among all genotypes. In NS3/4A, 18 positions were expected to be ubiquitinated whereas ten sites (136, 165, 210, 213, 360, 371, 372, 373, 551, 589) are conserved in genotypes (Table S8 highlighted in grey colour, Fig. 3; yellow rectangles). In NS5A, out of ten predicted high potential acetylation sites, only two sites at position 44 and 330 are found to be conserved among the genotypes (Table S8; highlighted in grey colour, Fig. 3; yellow rectangles). When ubiquitination sites have been predicted against HCV NS5B, 23 positions are found to have high potential for acetylation. Among these sites, only 12 are conserved in all major genotypes (Table S8; highlighted in grey colour, Fig. 3; yellow rectangles).

### Validation of PTMs

For confirming the PTMs and their effect on overall protein structure, conserved phosphorylation sites have been induced in the viral proteins and its effect is further srudied. It has been observed that by inducing phosphorylation in the viral proteins at a specific site, as predicted by Vienna-ptm softwares used in this study, they have been found to increase the stability of the protein. PDsum indicates that numbers of helices, sheets, beta hairpin, beta strands, gamma turns remarkably improve after the insertion of phosphorylation sites, confirming that these PTMs increase the stability of viral proteins, thus, increases their pathogenesis.

### Literature mining to investigate the role of HCV proteins in the development of HCC

Chronic hepatitis C virus (HCV) infection is one of the major causes of developing HCC. The natural course of HCV infection is fibrosis, cirrhosis, and hepatocellular carcinoma (HCC) which developed in a large number of HCV infected patients. It induces chronic inflammation in liver, which initiates several reactions of oxidative stress, steatosis, progressive fibrosis, cirrhosis and finally HCC. It is a multistep process and progresses over 20–40 years while each step represents a potential target for therapeutic intervention^15^. HCV related proteins also played a significant role in disturbing cell signaling finally affecting cell cycle and regeneration process. It is now reveled through various experimental evidences that HCC may result from a combined effect of many factors including host, environment and virus^15^. However immune mediated chronic inflammation during HCV infection may play a leading role in the development of HCC. Alteration in immune system induce HCC by changing many cellular pathways involved in key processes like cell proliferation, energy metabolism, and apoptosis. Among viral factors, different viral proteins played a critical role in the induction of HCC^12^. HCV core protein, which initiates HCV RNA replication, interacts with host proteins of cell-signaling pathways and modulate their interactions thus affecting overall immune response, oxidative stress, lipid metabolism and apoptosis^16,17^. It is a major regulator of steatosis through peroxisome proliferator-activated receptor alpha and sterol-regulatory element binding protein-1 pathways and it is considered as a major risk factor for the progression of HCC^18–20^. It interacts with ER or mitochondria and initiates ROS induced ER stress which alternatively causes DNA damage and speed up hepatocarcinogenesis progression. It is also involved in cell cycle processed like it play a critical role in G_1_/S transition by increasing the expression of cyclin E/Cdk2^21^ finally leading to apoptosis. It also showed interactions with tumor suppressor (P53, P73 and P21) and apoptosis regulators (TNF-α signaling or Bcl-2). It also played a key role in regulating growth and proliferation of cells by activating RAF/MAPK (Mitogen activated protein kinase)^22^ and Wnt/β-catenin, TGF-β pathways which are important regulators of HCC. NS3/4A protein interacts with tumor suppressor p53 and transform its ability. NS3 interact with interferon response factor (IRF-3) and inhibit induction of type-1 interferon leading to escape immune response. NS3/4A interacts with ATM and affect DNA repair mechanisms^23–25^. HCV NS5A is involved in replication, transcription factor activator and interacts with various signaling pathways including cell cycle/apoptosis, lipid metabolism and share common signaling targets with Core protein^12^. It inhibits apoptosis and mediate proliferation by targeting tumor necrosis factor-α, Bcl-2, PI3K, Wnt/β-catenin signal and mTOR. NS5A facilitate TGF-β to activate stellate cell causing fibrosis. NS5A affect EMT pathway and facilitate the transition of epithelial cells to mesenchymal stem cells^26,27^. NS5B causes proteasomal degradation of Rb protein^12,28^. It also activates E2F responsive genes leading to cell cycle progression. All these HCV proteins are involved in various steps of complex mechanism of carcinogenesis resulting in development of HCC. The above reports clearly demonstrate the role of HCV-related proteins on cellular pathways involved in progression of HCV induced HCC. The interplay of various reaction cascade between viral proteins and cellular pathways results in genomic imbalance thus disturbing the normal cellular reactions and leads to abnormal cell cycle and apoptosis. The interaction of viral proteins with various host cellular proteins, and the development of chronic liver disease during the course of HCV infection, represents a major role of viral proteins in the synchronization of chronic inflammation leading to the initiation and eventually progression of HCC. Although several lines of evidences are available to support the role HCV proteins in progression of HCC during HCV coarse of infection, however, much more analysis need to be done to extricate this mystery^12^.

In addition, numerous studies support the fact that MAPK plays a critical role in HCV replication as well as in progression of HCV induced HCC^29,30^. Moreover, p38 MAPK has also been previously reported to play a critical pro-viral role in the replication of several other viruses including influenza A virus^31,32^, HIV^33^, Human Cytomegalovirus^34^, Varicella-Zoster^35^, Chikungunya Virus^36^ and Junin virus^37^. On the other hand, inhibition of p38 signaling has been reported to impair replication of flaviviruses, respiratory viruses, enterovirus, rotaviruses, Chikungunya virus, SARS-CoV-2, junin virus as well as zika virus^37,38^. All these studies support the conclusion that these kinases are tightly involved in the positive regulation of HCV life cycle. It is evident that during HCV life cycle, MAPK pathway is activated that play the pivotal role in liver fibrosis and HCC progression^39,40^ as well as in viral immune evasion strategies occurring during HCV infection^41^. With regard to the role of MAPK cascades in HCV replication, virus binding to the CD81 receptor has been shown to activate the Raf/MEK/ERK pathway that was necessary for post-entry events^42^. Furthermore, the inhibitors of JNK and p38MAPK (SP600125 and SB203580 respectively) blocked HCV replication in replicon systems, suggesting that these kinases are tightly involved in positive control of HCV life cycle^43^. On the basis of all the above reported data it could be reasonably speculated that p38-MAPK pathway, could have a role both in early and late stages of HCV life cycle. Moreover, significantly high expression of p-p38 have been reported in the HCC patients with a larger tumor size^44^. In addition, it has also been reported from various evidences that HCV core, NS34A and NS5B proteins play a critical role in inducing oxidative stress that has been found to affect both virus replication as well as progression and severity of HCV infection^45^.

### Phosphorylation of HCV NS3/4A, NS5A and NS5B leads to HCV induced HCC

**Figure 4:**
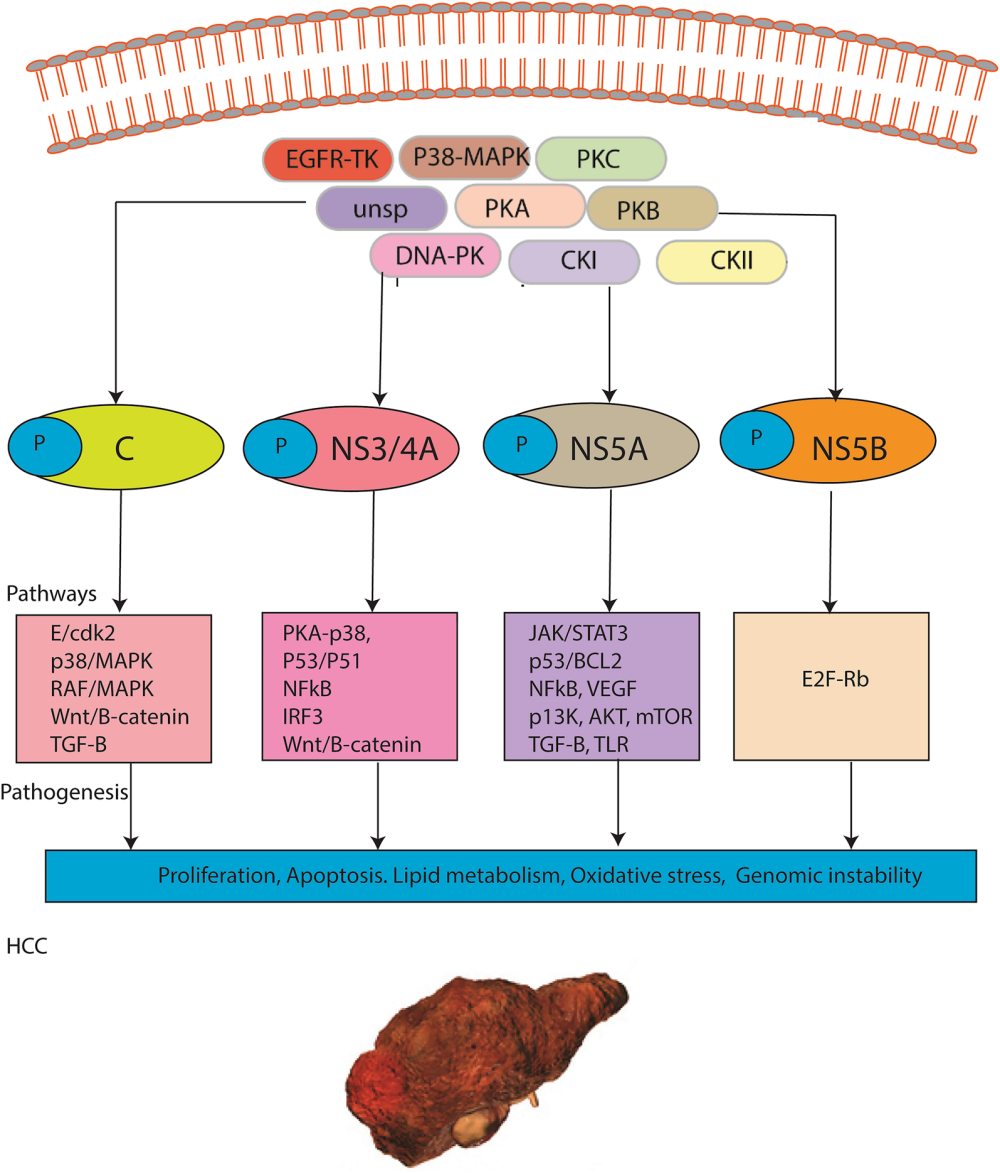
Possible mechanism adopted by HCV proteins to induce HCC. It shows the phosphorylating potential of various kinases (EGFR, p38-MAPK, PKC, unsp, PKA, PKB, DNA-PK, CKI and CKII) predicted through computation algorithms to phosphorylate HCV NS3/4A, NS5A and NS5B. This phosphorylation can support the viral proteins to initiate various pathways that lead to the development of HCC.

Post-translational modifications (PTMs) are involved in the variety of cellular activities and have also been shown to be involved in various types of cancers. A process of altered phosphorylation in many of the important cellular pathways is strongly associated with cancer. They serve as the potential targets for drug development against cancers. To investigate the role of phosphorylation of HCV Core, NS3/4A, NS5A and NS5B in HCC, we have observed possible kinases inducing phosphorylation in selected viral proteins using online Netphos and NetphosK servers. It has been observed that Unsp, p38-MAPK, cdk5, PKA, PKC, PKG, RSK, cdc2 are possible kinases to phosphorylate core protein while EGFR, unsp, CKI, PKC, PKA, PKB, PKC, p38MAPK, cdk5, cdc2, Cdk5, Gsk3, INSR, DNA-PK protein kinases are predicted to be phosphorylating HCV NS3/4A protein. In NS5A, cdc2, DMPK, PKC, EGFR, CKII, unsp, RSK, PKA, GSK3, p38MAPK, DNA-PK have been predicted to phosphorylate NS5A at different positions. While in NS5B, cdc2, CKI, PKC, unsp, p38MAPK, CKII, DNA-PK, PKA, PKG have been predicted to phosphorylate NS5B. All these predictions are predicted through advanced computational algorithms, Netphos and NetphosK. Furthermore, the role of these kinases in HCV induced HCC is investigated through literature mining. Based on this analysis, it has been observed that most of the predicted protein kinases phosphorylating the viral proteins play a critical role in HCC (Fig. 4), conferring the importance of these predicted sites. However, further experimental validations are needed to prove this data. HCV NS3/4A, NS5A, and NS5B are known players in HCV replication, pathogenesis and its progression towards HCC by regulating various cellular pathways including PKA/p38, p53/p21, NFkB, IRF3, Wnt/β-catenin (regulated by NS3/4A), JAK/STAT3, p53, BCL2, NFkB, VEGF, p13k/AKT/ mTOR, Wnt/β-catenin, TGF-β, TLR (regulated by NS5A) and E2F/Rb (NS5B). Most of these pathways are phosphorylation based activation pathways (Fig. 4) and their phosphorylation-dephosphorylation cascade has been shown to be involved in various types of cancers.

### Docking analysis with MAPK-p38

**Figure 5:**
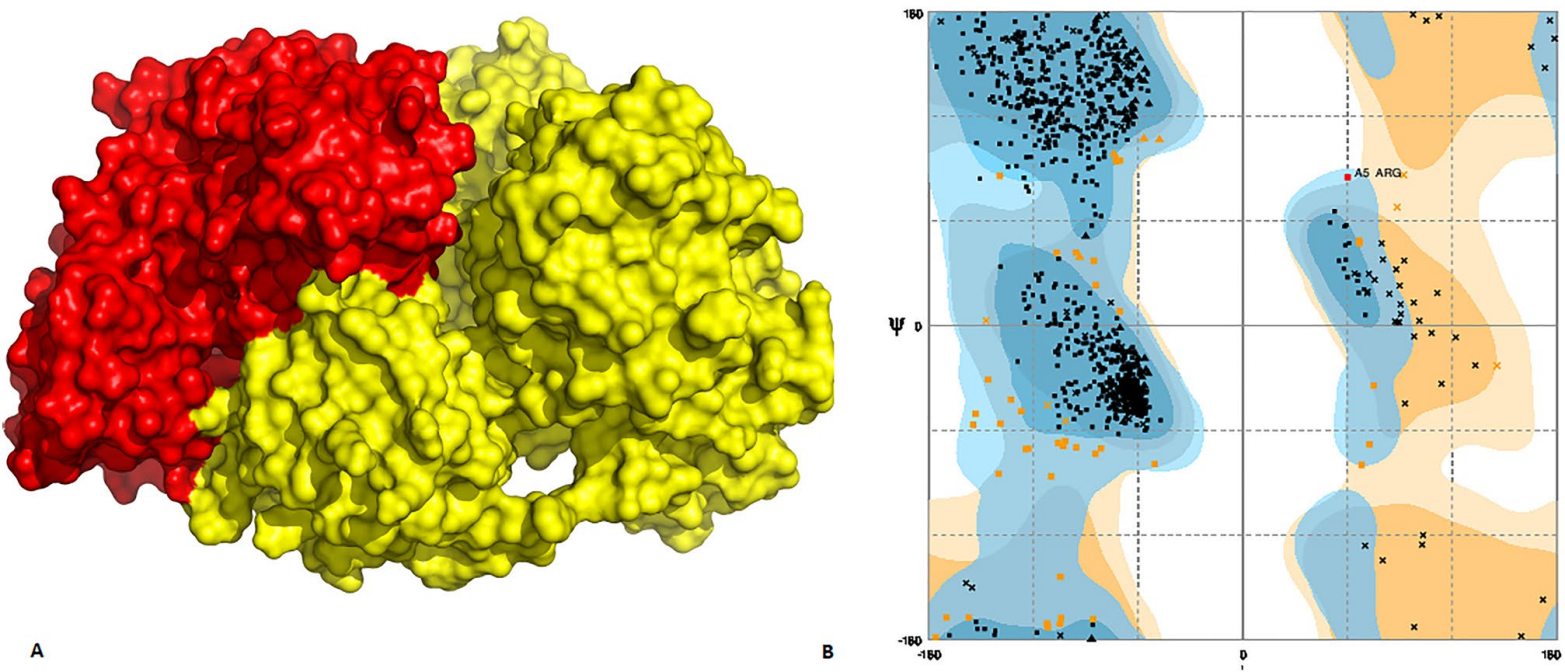
(**A**) Docked complex of HCV NS3/4A and p38 MAPK. Red color represents p38 MAPK while yellow signifies NS3/4A (**B**). Ramachandran plot shows allowed and disallowed regions.

**Figure 6:**
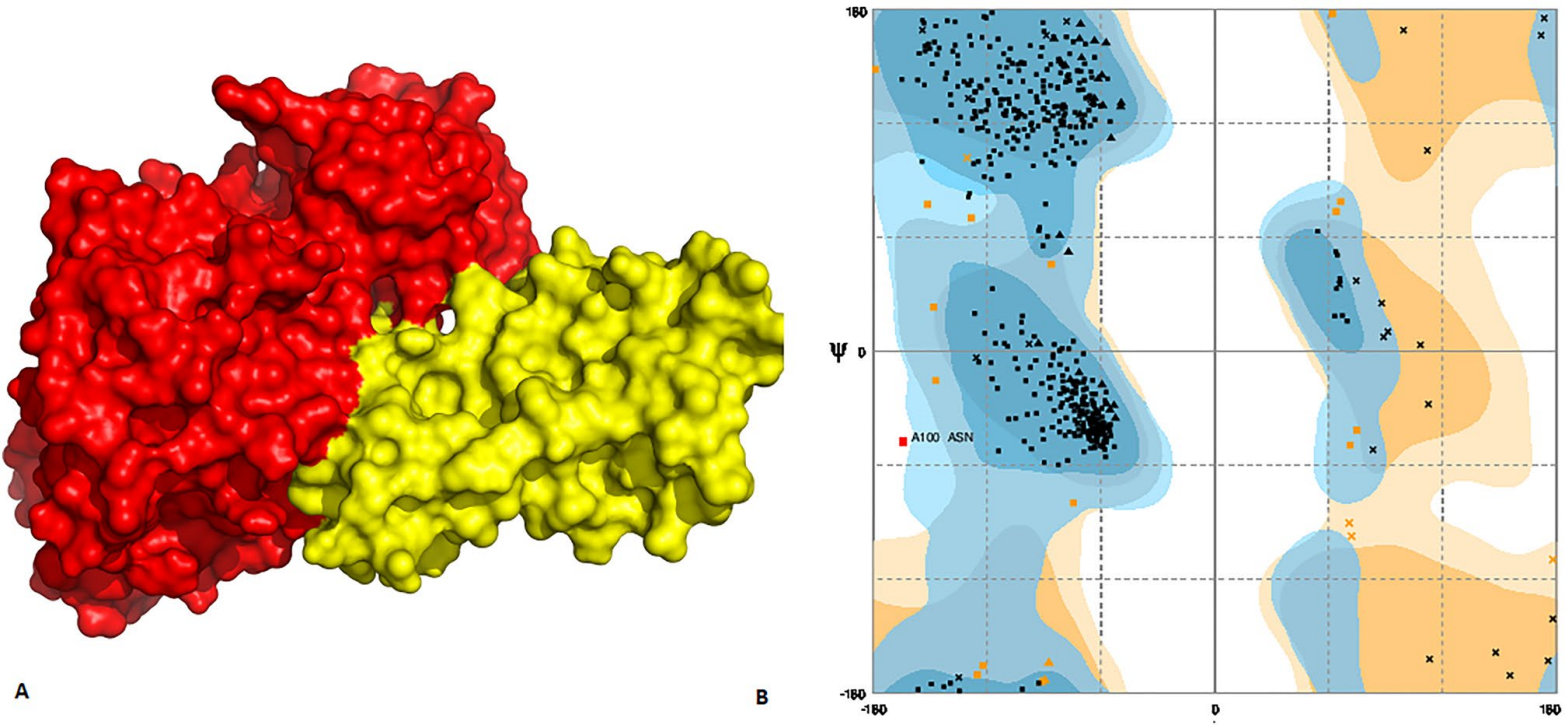
(**A**) Docked complex of HCV NS5A and p38 MAPK. Red color represents p38 MAPK while yellow signifies NS5A (**B**). Ramachandran plot shows allowed and disallowed regions.

**Figure 7:**
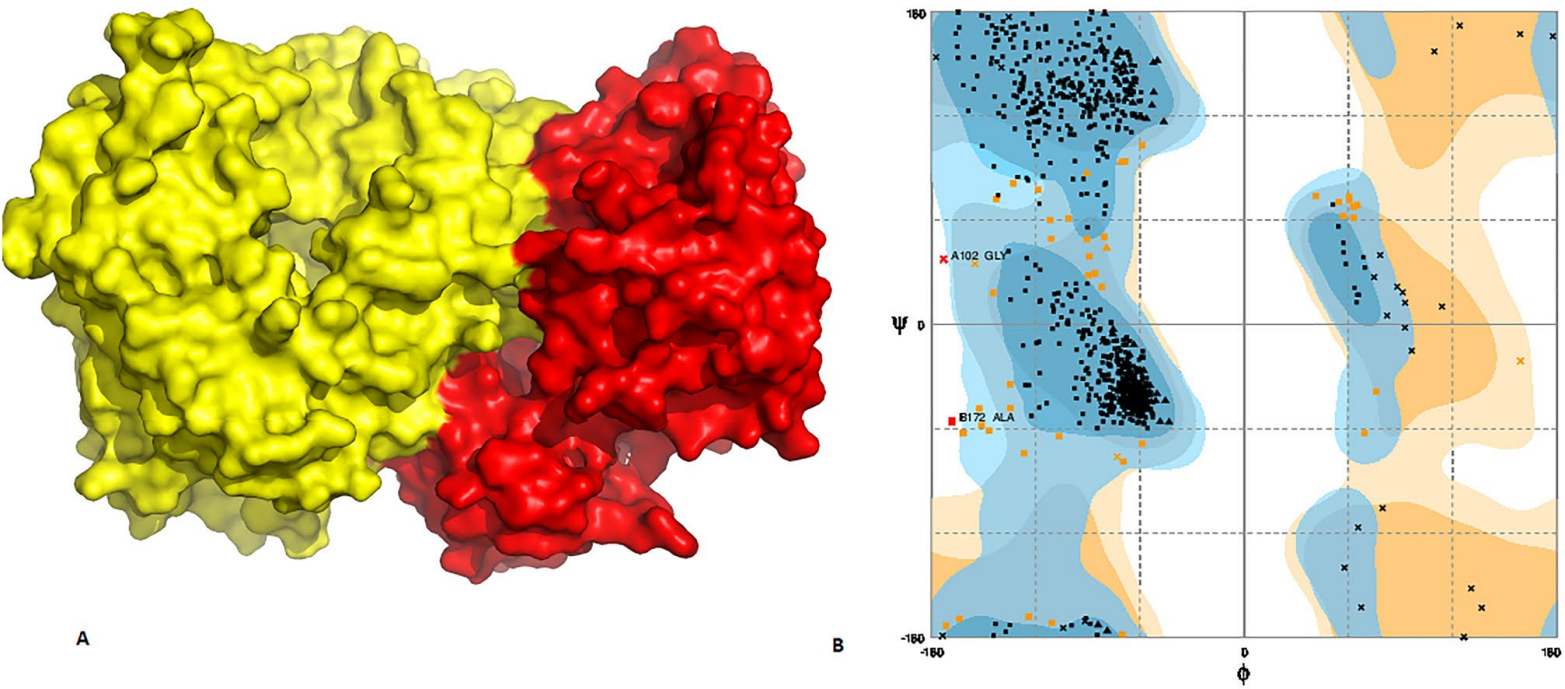
(**A**) Docked complex of HCV NS5B and p38 MAPK. Red color represents p38 MAPK while yellow signifies NS5B (**B**). Ramachandran plot shows allowed and disallowed regions.

Various protein kinases are responsible for cellular transduction signalling pathways and their malfunction or hyperactivity results in the development of several cancers. For instance, kinase signaling pathways have been shown to drive many of the hallmark phenotypes of cancer biology, for that they include proliferation, survival, motility, metabolism, angiogenesis and evasion of anti-cancer immune responses. Among these kinases, p38-MAPK cascades are tightly linked to oncogenic transformation and is a potential target for the development of anti-cancerous compounds^46^. Through extracellular stimulations, MAPKs are triggered by upstream MKKs that increase the phosphorylation of MAPKs. Subsequently, activated MAPKs phosphorylate target substrates more specifically on serine and threonine residues. p38-MAPKs is one of the sub family of MAPK, and is associated with HCC in many aspects^47^. To investigate the phosphorylating potential of P38-MAPK to HCV viral proteins (NS3/4A, NS5A and NS5B), docking analysis between NS3/4A, NS5A and NS5B and P38 MAPK has been performed by HADDOCK and only refined structures are selected for further evaluation. By applying the docking analysis, a strong association between three viral proteins and p38-MAPKs are observed. In case of NS3/4A, 14 strong hydrogen bonds are formed between NS3/4A and P38 MAPK (Fig. 5). In NS5A, 16 hydrogen bonds are formed while in NS5B 19 hydrogen bonds are observed (Figs. 6,7). This analysis confers strong relation between P38-MAPK and viral proteins suggests that P38-MAPK might play an important role in phosphorylating these proteins which, in turn, is important for activating cellular pathways lead to development of HCC in HCV infection.

### Validation of docked complex

For further validation of docked complex of HCV viral proteins and p38-MAPK, Ramachandran plot analysis has been performed. It is observed, in case of NS3/4A, 94.8% of the regions lied in favoured regions, 5.1% in allowed region and 0.1% in outlier regions (Fig. 5B). In NS5A, 95.7% of the structure comes in allowed region, 4.1 in favoured region while 0.2% in disallowed region (Fig. 6B). However, in NS5B, 94.7% of the structure is in allowed regions, 3.1% is in favoured regions and 0.2% is in disallowed regions. All these parameters confer the validity of docked complex structure (Fig. 7B).

## Discussion

HCV viral proteins interact with the host cellular proteins and different signaling pathways during HCV replication. Like cellular proteins, PTMs of HCV proteins play an integral role in protein functioning and regulation and, subsequently, affect the viral life cycle and making of new virus particles.They have been recognized as an important approach for modulating the activities of proteins^48^ and are traced as disease markers while many of them are used as important targets for emergent target-specific therapies^49^. It has been reported that when specific PTM site like phosphorylation site is mutated in HIV, it blocks the PTM site within the virus and results in decreased infectivity and defective maturation of viral particle. It is established that many viral and cellular kinases could serve as targets for both therapeutic and prophylactic treatments of viral infections. More specifically, Kinase-inhibitory compounds have previously been used in the treatment of various cancers, besides that a rigorous research is being continued to find out their abilities in treating viral infections^50^. Many PTMs dysregulation are known to be the major cause of development of cancers including HCV induced HCC. Various PTMs associated pathways including p38-MAPK, DNA-PK, CKI, II,II plays a vital role in HCV pathogenesis that leads to the development of HCC^51^. Moreover, these dysregulated pathways have shown the strong association with HCV viral proteins including NS3/4A, NS5A and NS5B. In support of this, the above mentioned PTMs have been reported in HCV drug targeting proteins (C, NS3/4A, NS5A and NS5B) and are known to be involved in affecting the key cellular processes^7^, as shown in Fig. 1. Furthermore, HCV viral protein expressions are involved in deregulation of the mitotic checkpoint and expression of chemokine IL-8 through p38-dependent pathway, which largely accounts for the initiation stage of hepatocarcinogenesis. These studies illustrate that PTMs are important in various cellular processes, which are involved in HCV progression towards HCC. This is one potential reason of hijacking by HCV to strengthen the mechanism of its existence within the host cells.

Comprehensive studies on the presence of PTMs in these viral proteins and their possible association with HCV induced HCC is still lacking. Many speculations explaining the phenomenon of HCV proteins’ PTMs and their association with HCC are not able to have consensus on the precise mechanism followed by the pathogen. Therefore, in the current study, we have prioritized highly potential PTM sites within the HCV drug target proteins and have investigated their possible mechanism of inducing HCC.

**Figure 8:**
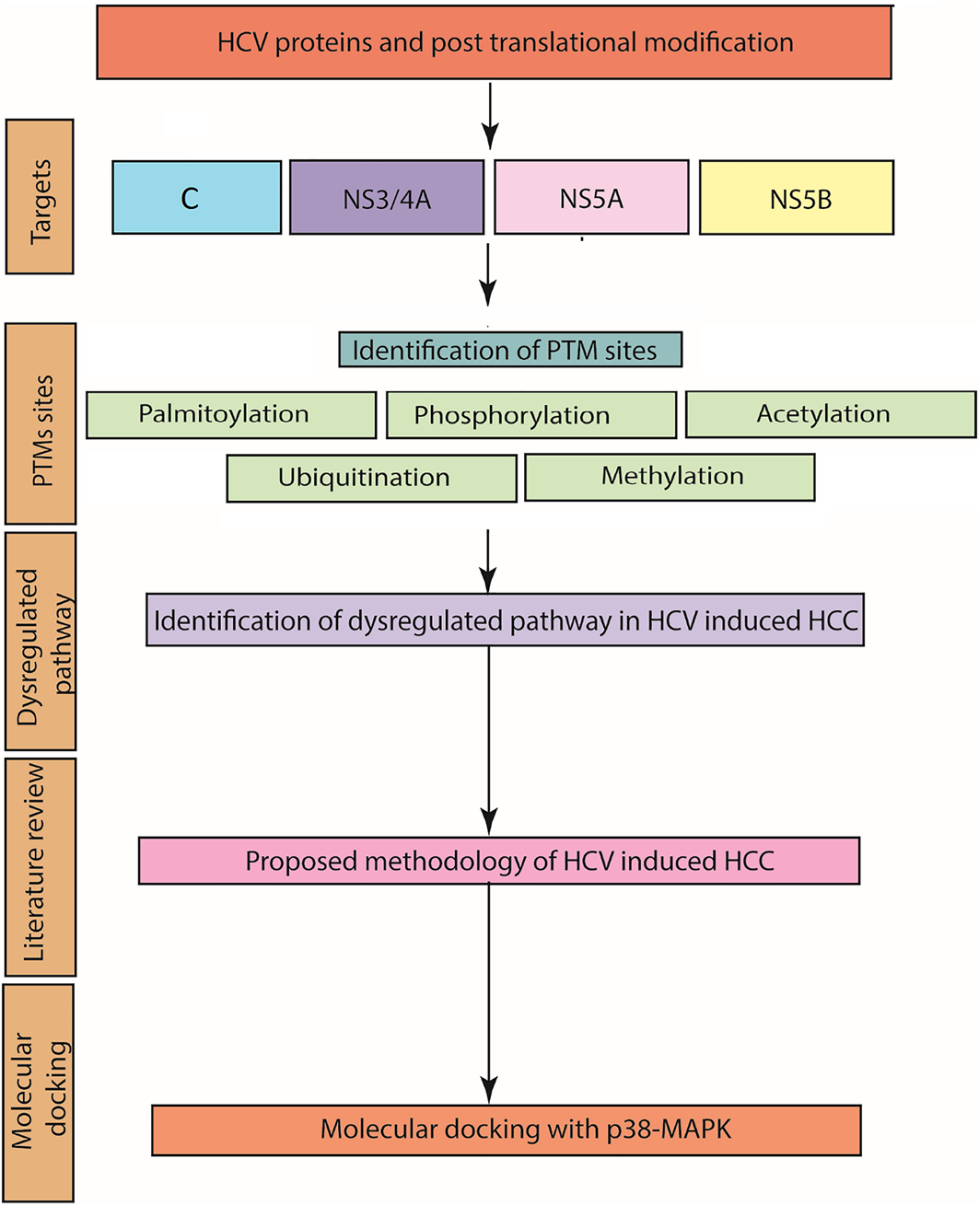
Overview of methodology adopted in our study.

PTM sites identified within HCV C, NS3/4A, NS5A and NS5B proteins in this study have highlighted some key sites that can be targeted by the HCV to dysregulate the normal functioning of proteins involved in various cellular pathways. All predicted sites are highly conserved that signifies their evolutionary aspect. For further confirmation of PTMs of HCV viral proteins involved in HCV induced HCC, docking has been performed between HCV NS3/4A, NS5A, NS5B and p38-MAPK. The deregulated p38-MAPKs are often found to play a significant role in the development of HCC^47^. Docking analysis has also revealed strong association between p38-MAPK that necessitates the additional experimental validation to confirm their destructive effect. Some of the PTMs predicted in our analysis are also experimentally validated. HCV NS3/4A is known to be methylated at Arg1493 in the 1486Gln-Arg-Arg-Gly-Arg-Thr-Gly-Arg-Gly1494 motif by PRMT1^52^. In our analysis this site was predicted to be highly methylated and was found to be conserved among all major genotypes conferring the importance of integrated informatic analysis (Fig. 2). HCV NS5A is known to be highly phosphorylated protein and is required for RNA replication and virion morphogenesis. The basally phosphorylated sites are mainly serine and threonine residues that are known to be in the central and C-terminal regions. All the possible phosphorylation sites in NS5A has been analysed in our study and it is observed that most of these sites are either present in the middle or in the C terminal regions (Fig. 8). Serine 225 in NS5A has been reported to be a phosphorylation site and its mutations resulted in reduction of genomic replications^53^. This important phosphorylation site was also predicted to be phosphorylated and is conserved among almost all genotypes (1–7) (Fig. 8, Table S2). Also, a number of kinases (CKI and CKII) are involved in phosphorylating HCV NS5A protein and contributes to NS5A hyper-phosphorylated form. These sites may be contributing in shifting the viral protein from genome replication to particle assembly^54^. Many putative CKI and CKII mediated phosphorylated sites are also predicted in our study. PTMs also have the potential of regulating viral RNA-dependent-RNA polymerase through phosphorylation. It is also suggested that NS5B phosphorylation can increase HCV replication^55^. We have predicted highly potential phosphorylation sites among NS5B that could be the target for further studies. There is also association between HCV NS5B and hPLIC1 (human homolog 1 of protein linking integrin-associated protein and cytoskeleton). This interaction ratifies ubiquitin based proteasome degradation that results in decreased level of NS5B^56^. As the ubiquitination sites within NS5B are still not clear, therefore, detailed information is required. In our study, we have attempted to explore all possible ubiquitination sites within NS5B.

## Materials and methods

The current study has been characterised into three main parts, 1: scrutinization of HCV C, NS3/4A, NS5A, NS5B proteins for potential conserved PTM sites that can be induced by the virus during its coarse infection.2: Characterising the role of these PTMs towards the progression of HCV induced HCC, 3: Docking analysis between MAPK-P38 with NS3/4A, NS5A and NS5B to investigate the potential role of p38-MAPK in phosphorylating HCV proteins. A summary of the approach followed in the present study is shown in Fig. 8.

### Data integration

Consensus sequences of available protein sequences of HCV NS3/4A (1810), NS5A (1700), NS5B (1350) were retrieved from the sequences retrieved from Genbank. These sequences were taken against all major genotypes 1–7 (Reference sequences: GenBank accession numbers: HCV-1a, NC_004102; HCV-1b, AJ238799; HCV-2a, NC_009823; HCV-2b, D10988; HCV-2c, D50409; HCV-3a, NC_009824; HCV-3b, D49374;HCV-4, NC_009825; HCV-5, NC_009826; HCV-6, NC_009827; HCV-7,EF108306). The sequences from each genotype were first aligned to obtain the consensus sequence and, subsequently, the achieved consensus sequences were aligned with each other to secure a global consensus sequence (Supplementary Fig. 1).

### Prediction of sites prone to post-translational modifications

Various alteration sites including kinase specific phosphorylation, acetylation, palmitoylation, ubiquitination, methylation and Ying-Yang have been predicted within HCV C,NS3/4A, NS5A and NS5B.

#### Prediction of possible phosphorylation sites

Several HCV C, NS3/4A, NS5A and NS5B phosphorylation sites have been reported but still many other of these sites needs to be explored^57–59^. Netphos 2.0^60^, which is an artificial neural network based program, is used for the prediction of putative phosphorylation sites of HCV C, NS3/4A, NS5A and NS5B for each Thr, Ser and Tyr residues with minimum threshold value of 0.5. NetPhosK 1.0^61^ has been used for the prediction of kinase-specific phosphorylation sites in selected viral proteins. Conservation analysis of these sites among all genotypes (1–7) is also performed.

#### Prediction of acetylation and palmitoylation sites

Prediction of acetylation on internal lysine (PAIL) server^62^ has been used to identify potential acetylation sites within HCV C, NS3, NS5A and NS5B. Conservational pattern of these sites among all genotype has been also investigated. CSS-PALM 3.0^63^, an online server, has been used to predict possible palmitoylation sites. This server predicts several potential sites based on experimentally proved palmitoylation reported within many proteins.

#### Prediction of methylation sites

Some sites in HCV non-structural proteins including C, NS3, NS5A and NS5B have been reported to be methylated sites. For further confirmation of putative methylated sites, PMes program^64^ has been used. PMesis based on improved feature encoding scheme and support vector machine. This prediction server provides better prophetic results when are compared to other predicting servers. Conserved methylation sites among all genotypes have also been explored.

#### Prediction of ubiquitination sites

For exploration of ubiquitination sites within the selected viral proteins UBpred tool has been^65^. It predicts potential ubiquitination sites in proteins based on random forest-based prediction. Conservation pattern among all genotype has also been investigated.

### Identification of surface distribution of PTMs

PDB structures of NS3/4A (1CU1), NS5B (3QGH) has been used to study surface distributions of conserved PTMs. For C and NS5A, PDB structure has been predicted by I-Tasser and has been used to analyse the conserved PTMs distribution.

### Confirmation of PTMs sites

To confirm the role of PTMs sites and their potential effects, conserved phosphorylation has been induced in the NS5B by using Vienna-ptm (29). The confirmation and stability of the viral protein before and after the phosphorylation induction has been checked by using PDBsum server (30).

### Role of PTMs in HCV induced HCC

Identifying kinase substrate and their related phosphorylation sites is important to expose molecular mechanism of particular disease development. To investigate the role of PTMs in HCV induced HCC, we find out the possible protein kinases involved in the phosphorylation of HCV viral proteins C, NS3/4A, NS5A and NS5B. For this the phosphorylation inducing kinases, which have been predicted to be involved in phosphorylating selected viral proteins, have been screened out by using Netphos and NetphosK. Further literature mining has been performed to find the possible role of predicted protein kinase in HCV induced HCC. In-depth literature mining of the experimental studies assist in making logic based figure of HCV proteins phosphorylation aided HCV progression towards HCC. It represents important phosphorylation specific signalling pathways, triggered by HCV C, NS3/4A, NS5A and NS5B that could lead towards the progression of HCC.

### Docking of viral proteins with P38 MAPK

Deregulated p38-MAPKexpressions have been associated with various cancer development including HCC. Phosphorylate P38 has been observed in many HCV induced HCC patients^44,66^. HCV Core Protein stimulates ERK and p38-MAPK in the presence of Ethanol in Transgenic Mice^22^. In addition p-p38 levels are significantly higher in the HCC patients with a larger tumor^44^. To investigate the possible role of P38-MAPK in phosphorylating the HCV Core, NS3/4A, NS5A and NS5B, docking analysis has been performed. High Ambiguity Driven protein–protein docking (HADDOCK) is used for docking between P38 MAPK and HCV NS3/4A, NS5A and NS5B. The available 3D structures of HCV Core (Predicted by I-Tasser), NS3/4A (4A92), NS5A (3FQM) and NS5B (3MWV) are used while for p38 MAPK, 3D structure 1A9U has been employed.

## Supplementary Information

Supplementary Information 1.

Supplementary Information 2.

Supplementary Information 3.

## Data Availability

The authors declare that the data supporting the findings of this study are addressed within the article and confirm that all the datasets analyzed during the current study are accessible from NCBI nucleotide; https://www.ncbi.nlm.nih.gov/nucleotide, Netphos; http://www.cbs.dtu.dk/services/NetPhos/, NetPhosK; http://www.cbs.dtu.dk/services/NetPhosK/, PAIL; http://bdmpail.biocuckoo.org/, CSS-PALM 3; http://csspalm.biocuckoo.org, PMes program; http://bioinfo.ncu.edu.cn/inquiries_PMeS.aspx, UBpred; http://www.ubpred.org/, I-TASSER; https://zhanggroup.org/I-TASSER/, vienna-ptm; http://vienna-ptm.univie.ac.at/, PDBsum; http://www.ebi.ac.uk/thornton-srv/databases/pdbsum/.
