# Supplementary material for: Integrated analysis to study the interplay between post-translational modifications (PTM) in hepatitis C virus proteins and hepatocellular carcinoma (HCC) development
Source: Sci Rep. 2022 Sep 19;12:15648. doi: 10.1038/s41598-022-19854-6 (PMC9483894; doi:10.1038/s41598-022-19854-6)
Supplement: Supplementary file 1 — Supplementary Information 1. [file 41598_2022_19854_MOESM1_ESM.docx]

**Supplementary figure 1:**

**Legend:**

Graphical illustration of global consensus sequence of HCV Genotypes
