# Supplementary figures and images for: Integrated analysis to study the interplay between post-translational modifications (PTM) in hepatitis C virus proteins and hepatocellular carcinoma (HCC) development
Source: Sci Rep. 2022 Sep 19;12:15648. doi: 10.1038/s41598-022-19854-6 (PMC9483894; doi:10.1038/s41598-022-19854-6)

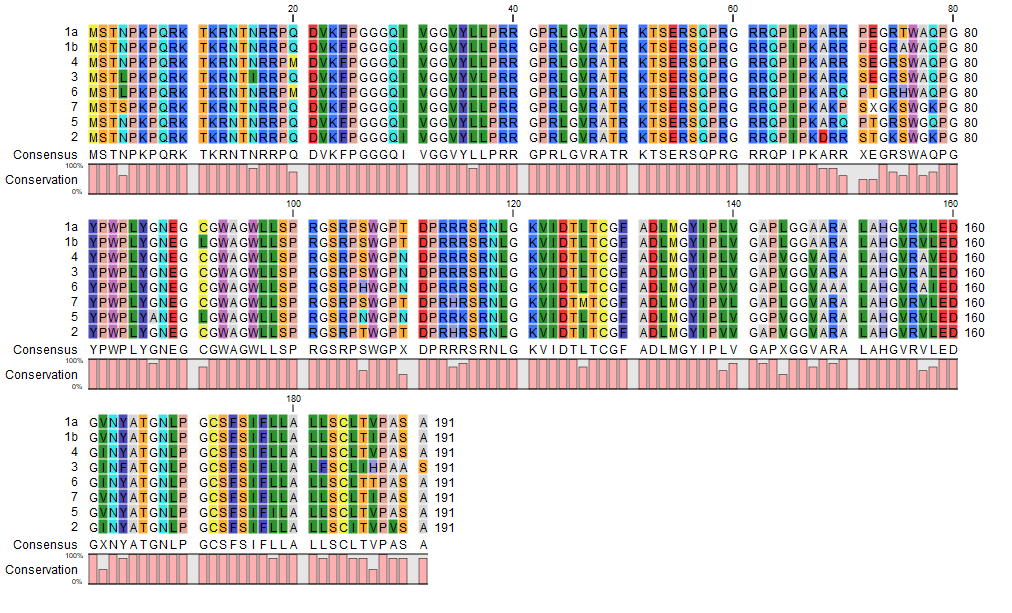

Supplement: Supplementary file 2 — Supplementary Information 2. [file 41598_2022_19854_MOESM2_ESM.png]
