# Supplementary material for: Integrated analysis to study the interplay between post-translational modifications (PTM) in hepatitis C virus proteins and hepatocellular carcinoma (HCC) development
Source: Sci Rep. 2022 Sep 19;12:15648. doi: 10.1038/s41598-022-19854-6 (PMC9483894; doi:10.1038/s41598-022-19854-6)
Supplement: Supplementary file 3 — Supplementary Information 3. [file 41598_2022_19854_MOESM3_ESM.docx]

Table S1-S4:

Predicted phosphorylation sites in HCV C, NS3/4A, NS5A and NS5B. Conserved phosphorylation sites are highlighted in pink (Table S1; core) yellow (Table S1; NS3/4A), orange (Table S2; NS5A) and green (Table S3; NS5B).

| Table S1 | | | | |
| --- | --- | --- | --- | --- |
| Amino Acids Name | **Position** | Conservation among genotypes | kinases | Structural localisation |
| Ser | 2 | Y | PKC | E |
| Thr | 3 | Y | PKC | E |
| Thr | 11 | Y | PKC,uncp | E |
| Thr | 15 | Y | Unsp,PKC | E |
| Thr | 49 | Y | PKC, cdc2, unsp | E |
| Thr | 52 | Y | Unsp, PKC | E |
| Ser | 53 | Y | Unsp, RSK, PKC, PKG | E |
| Ser | 56 | Y | Unsp, DNAPK | E |
| Ser | 75 | Y | Unsp | E |
| Tyr | 86 | Y | Unsp | E |
| Ser | 99 | Y | Unsp, p38-MAPK, cdk5 | B |
| Ser | 103 | Y | PKA, Unsp | B |
| Thr | 110 | Y | Cdk5 | E |
| Ser | 116 | Y | Unsp, PKA, PKG, RSK | E |
| Thr | 125 | Y | Cdc2 | E |
| Thr | 166 | Y | Cdc2 | E |
| Ser | 173 | Y | PKA, cdc2 | E |
| Ser | 175 | Y | Cdc2 | B |
| Thr | 186 | Y | Cdc2 | B |

| Table S2 | | | | |
| --- | --- | --- | --- | --- |
| Amino Acids Name | **Position** | **Conservation among genotypes** | **kinases** | **Structural localisation** |
| Tyr | 6 | Y | EGFR | B |
| Thr | 10 | Y | unsp | E |
| Thr | 16 | N | CKI | B |
| Ser | 20 | Y | CKI | B |
| Thr | 22 | Y | Unsp  PKC | B |
| Thr | 38 | Y | PKC | E |
| Thr | 40 | N | DNAPK | B |
| Ser | 61 | N | PKC | E |
| Tyr | 75 | N | Unsp  EGFR | B |
| Ser | 93 | Y | PKA | E |
| Thr | 95 | N | P38MAPK  Cdk5 | E |
| Ser | 101 | N | PKA  Cdc2 | E |
| Tyr | 105 | N | Unsp | B |
| Ser | 122 | N | Unsp  PKA  PKB  RSK | E |
| Ser | 125 | N | Unsp  Cdc2  PKA | B |
| Ser | 128 | Y | Unsp  P38MAPK  PKC | B |
| Ser | 133 | N | Unsp  Cdc2 | E |
| Thr | 134 | N | Unsp  PKC | B |
| Ser | 138 | N | Unsp  Cdc2 | E |
| Ser | 139 | Y | Unsp  PKA | E |
| Thr | 160 | N | PKC | E |
| Thr | 177 | N | PKC | E |
| Thr | 178 | N | unsp | E |
| Ser | 181 | Y | Unsp  Cdc2  Cdk5  Gsk3 | B |
| Ser | 188 | Y | Cdc2 | E |
| Ser | 189 | N | P38MAPK  Cdk5 | E |
| Ser | 208 | Y | Unsp  PKC | E |
| Ser | 211 | Y | Unsp  PKC | B |
| Thr | 212 | Y | Unsp | B |
| Thr | 260 | Y | Unsp | E |
| Thr | 261 | Y | PKC | B |
| Thr | 266 | Y | CKI | B |
| Ser | 268 | Y | PKC | B |
| Thr | 269 | Y | PKC | B |
| Tyr | 270 | Y | INSR | B |
| Tyr | 284 | Y | Unsp | B |
| Ser | 294 | N | CKII | B |
| Tyr | 295 | N | CKII | B |
| Thr | 305 | Y | CKII | B |
| Thr | 324 | Y | Cdk5  P38MAPK  Unsp  GSK3 | B |
| Ser | 328 | Y | PKC  Cdc2 | E |
| Thr | 343 | N | PKC  CKI  CKII | B |
| Tyr | 350 | Y | EGFR | E |
| Ser | 370 | Y | PKC | E |
| Ser | 398 | Y | Unsp | E |
| Thr | 402 | Y | Unsp | E |
| Ser | 424 | Y | Unsp | B |
| Thr | 435 | N | PKC | E |
| Ser | 439 | Y | DNAPK | E |
| Thr | 445 | N | Unsp | E |
| Thr | 448 | Y | PKC | B |
| Ser | 457 | Y | Unsp | B |
| Ser | 459 | N | Unsp  ATM  DNAPK | E |
| Ser | 483 | Y | Unsp  PKA | E |
| Ser | 489 | N | Cdc2  CkII | B |
| Thr | 505 | N | Unsp | E |
| Thr | 510 | N | Unsp  PKC | E |
| Thr | 519 | N | GSK3  P38MAPK | B |
| Thr | 537 | Y | CKII | E |
| Thr | 540 | Y | PKC | E |
| Ser | 548 | Y | ATM  DNPK | E |
| Thr | 559 | Y | unsp | B |
| Thr | 596 | y | CDK5  P38MAPK | B |
| Thr | 612 | Y | PKC | B |
| Thr | 651 | N | PKC | B |
| Ser | 663 | N | Unsp  PKC | E |
| Tyr | 676 | Y | unsp | E |

|  | | Table S3 |  |  |
| --- | --- | --- | --- | --- |
| Amino acids  Name | | **Conservation among genotypes** | **kinases** | **Structural localisation** |
|  | **Position** |  |  |  |
| Ser | 3 | N | PKC  Cdc2 | E |
| Thr | 21 | N | PKC | E |
| Thr | 71 | N | PKC  DNAPK | E |
| Ser | 81 | N | Cdc2 | B |
| Thr | 95 | N | PKC | B |
| Ser | 98 | N | Cdc2 | B |
| Tyr | 106 | Y | Unsp | B |
| Ser | 114 | N | Unsp  CKII | B |
| Tyr | 118 | Y | Unsp  EGFR | B |
| Thr | 151 | N | Unsp | E |
| Ser | 174 | N | Cdc2 | B |
| Ser | 176 | N | Cdc2 | B |
| Ser | 186 | Y | DNAPK | E |
| Ser | 207 | Y | Unsp | E |
| Thr | 213 | N | PKC | B |
| Ser | 222 | Y | Unsp  Cdk5  P38MAPK  PKA  GSK3 | E |
| Ser | 225 | Y | Unsp  PKC  Cdc2 | E |
| Ser | 228 | Y | PKC  Cdc2 | E |
| Ser | 230 | Y | Cdc2 | E |
| Ser | 232 | Y | Unsp  DNAPK  PKC | E |
| Ser | 235 | Y | Unsp | E |
| Ser | 238 | Y | Unsp  PKC | E |
| Thr | 244 | N | PKC | E |
| Thr | 245 | N | PKC | E |
| Thr | 270 | N | CKII | E |
| Ser | 274 | N | Unsp  RSK | E |
| Ser | 297 | Y | Unsp  PKA  RSK | E |
| Thr | 328 | N | PKC | E |
| Tyr | 334 | N | Unsp | B |
| Thr | 360 | N | PKA  PKG  unsp | B |
| Thr | 367 | N | Cdc2 | E |
| Ser | 369 | N | unsp | B |
| Thr | 377 | Y | Cdc2 | E |
| Ser | 379 | N | PKC  CKI | B |
| Ser | 383 | N | Unsp | E |
| Ser | 384 | N | Unsp  Cdc2 | E |
| Ser | 387 | N | Unsp | E |
|  | 389 | N | Unsp  Cdc2  CKII | E |
| Ser | 391 | N | Unsp  Cdc2  CKII | E |
| Ser | 397 | N | Cdc2 | E |
| Ser | 400 | N | Unsp  Cdk5  P38MAPK | E |
| Ser | 412 | N | Unsp  PKC  CKII | B |
| Ser | 414 | N | Unsp  PKC | B |
| Ser | 417 | N | Unsp | E |
| Ser | 431 | Y | Unsp | E |
| Ser | 432 | Y | Unsp  PKC  Cdc2 | E |
| Ser | 434 | Y | CKI  Cdc2 | E |
| Thr | 437 | N | Unsp  CKII  PKC | E |
| Ser | 439 | N | Unsp  CKII | E |
| Ser | 441 | N | CKII | E |

|  | | Table S4 |  |  |
| --- | --- | --- | --- | --- |
| Amino Acids | | **Conservation among genotypes** | **kinases** | **Structural localisation** |
| Name | Position |  |  |  |
| Ser | 3 | Y | Cdc2 | E |
| Ser | 5 | N | CKI | B |
| Thr | 7 | Y | PKC | B |
| Thr | 12 | Y | Unsp  P38MAPK | E |
| Ser | 29 | N | Cdc2 | E |
| Ser | 39 | N | Cdc2 | B |
| Thr | 40 | Y | Cdc2 | B |
| Thr | 41 | N | Unsp  PKC | E |
| Ser | 42 | Y | PKC | B |
| Thr | 53 | Y | PKC | E |
| Ser | 62 | N | Unsp | E |
| Tyr | 64 | Y | Unsp | B |
| Ser | 76 | Y | PKC | E |
| Ser | 84 | N | Unsp  CKII | B |
| Thr | 92 | N | Cdk5  GSK3  P38MAPK | B |
| Ser | 96 | Y | Unsp  PKC | E |
| Ser | 99 | Y | PKC  Unsp | E |
| Tyr | 103 | N | Unsp | B |
| Ser | 130 | N | Unsp | E |
| Thr | 137 | Y | PKC | B |
| Ser | 189 | N | Cdc2 | E |
| Ser | 196 | Y | Cdk5  P38MAPK | E |
| Ser | 210 | N | PKC | E |
| Thr | 213 | N | Unsp  P38MAPK | E |
| Ser | 226 | Y | CKII | E |
| Thr | 227 | Y | CKII | E |
| Thr | 235 | N | CKII | B |
| Ser | 255 | N | Unsp  DNAPK  PKA | B |
| Thr | 257 | Y | PKC  Unsp | B |
| Ser | 282 | Y | Unsp  PKA | B |
| Thr | 287 | Y | PKC | B |
| Ser | 288 | Y | Unsp | B |
| Thr | 292 | Y | CKI | B |
| Thr | 340 | N | Unsp | E |
| Tyr | 346 | Y | Unsp | B |
| Ser | 347 | Y | Unsp  PKA  PKG | B |
| Ser | 365 | Y | Cdc2 | B |
| Ser | 367 | Y | Cdc2 | B |
| Ser | 368 | Y | Cdc2 | B |
| Ser | 377 | N | Unsp | E |
| Thr | 385 | Y | Uncp  PKC | B |
| Thr | 390 | N | P38MAPK  CDK5  Unsp | E |
| Thr | 399 | Y | Unsp  PKC | B |
| Thr | 403 | N | Unsp  P38MAPK  GSK3 | E |
| Thr | 427 | Y | PKC | B |
| Ser | 435 | N | Unsp  DNAPK  ATM  CDKII | E |
| Thr | 451 | N | PKC | B |
| Ser | 453 | N | PKA | E |
| Ser | 473 | N | Unsp  Cdc2  CKI | B |
| Ser | 476 | N | Unsp  Cdc2 | B |
| Ser | 478 | Y | Unsp | E |
| Thr | 532 | Y | PKC | E |
| Thr | 537 | Y | P38MAPK | E |
| Thr | 552 | N | PKC | B |
| Ser | 556 | N | Unsp  Cdc2 | E |
| Tyr | 561 | N | Unsp | B |
| Ser | 563 | Y | Unsp  Cdc2 | B |
| Ser | 565 | N | Unsp | E |

Table S5-S6:

Predicted acetylation and palmitoylation sites in HCV C, NS3/4A, NS5A and NS5B. Conserved sites are highlighted in blue colour (Table S5), pink colour (Table S6).

|  | Table S5 |  |
| --- | --- | --- |
| Peptide(Core) | **Position** | **Conservation** |
| MSTNPKPQRKTK | 6 | Y |
| NPKPQRKTKRNTN | 10 | Y |
| KPQRKTKRNTNRR | 12 | Y |
| RRPQDVKFPGGGQ | 23 | Y |
| GVRATRKTSERSQ | 51 | Y |
| RRQPIPKARRPEG | 67 | Y |
| Peptide(NS3/4A) | **Position** | **Conservation** |
| YHGAGSKTLAGPK | 62 | N |
| KTLAGPKGPVXQM | 68 | N |
| PAPPGAKSLTPCT | 92 | N |
| RPISTLKGSSGGP | 136 | Y |
| CTRGVAKAVDFVP | 165 | Y |
| APTGSGKSTKVPA | 210 | Y |
| GSGKSTKVPAAYA | 213 | Y |
| TYSTYGKFLADGG | 272 | Y |
| LIFCHSKKKCDEL | 371 | Y |
| IFCHSKKKCDELA | 372 | Y |
| FCHSKKKCDELAA | 373 | Y |
| CDELAAKLXXLGL | 380 | N |
| KCLIRLKPTLHGP | 589 | Y |
| RIVLSGKPAVIPD | 665 | N |
| Peptide(NS5A) |  |  |
| TVLSDFKTWLKAK | 20 | N |
| KTWLKAKLLPXLP | 26 | Y |
| SCQRGYKGVWRGD | 44 | N |
| RFAPPCKPLLRXE | 166 | N |
| LSAPSLKATCTTH | 240 | N |
| VPPPRRKRTVVLT | 358 | Y |
| LAELAEKSFGQSS | 378 | N |
| Peptide (NS5B) | **Position** |  |
| CAAEEEKLPINPL | 20 | N |
| SASQRQKKVTFDR | 50 | Y |
| ASQRQKKVTFDRL | 51 | Y |
| DVLKEXKARASXV | 72 | Y |
| ARASXVKARLLSV | 79 | N |
| PHSARSKFGYGAK | 100 | Y |
| KFGYGAKDVRSLS | 106 | Y |
| VRSLSSKAXNHIN | 114 | N |
| HINSVWKDLLEDS | 124 | N |
| FCVDPEKGGRKPA | 151 | Y |
| PEKGGRKPARLIV | 155 | Y |
| GVRVCEKRALYDX | 172 | Y |
| FLLKAWKSKKTPM | 209 | N |
| LKAWKSKKTPMGF | 211 | Y |
| KAWKSKKTPMGFS | 212 | N |
| LDPEARKAIXSLT | 251 | N |
| TLTCYIKAXAACR | 298 | N |
| AHDASGKRVYYLT | 379 | N |
| VAXCLRKLGVPPL | 491 | N |
| ARAVRAKLIAQGG | 510 | N |
| RAAICGKYLFNWA | 523 | N |
| AVXTKLKLTPLPA | 535 | N |

|  | Table S6 |  |
| --- | --- | --- |
| Position | **Peptide** | **Conservation** |
| NS5A | |  |
| 13 | RDXWDWV**C**TVLSDFK | Y |
| 39 | PGXPFLS**C**QRGYKGV | Y |
| 342 | XPPVVHG**C**ALPPPXX | Y |
| NS5B | |  |
| 14 | TGALITP**C**AAEEEKL | N |
| 170 | PDLGVRV**C**EKRALYD | Y |
| 242 | TEEXIYQ**C**CDLDPEA | N |
| 274 | XNSKGQX**C**GYRRCRA | N |

Table S7:

Predicted methylation sites in HCV C, NS3/4A, NS5A and NS5B. Conserved sites are highlighted in purple colour (Table S7).

|  |  | Table S7 |  |
| --- | --- | --- | --- |
| Protein name | **Position of site** |  | **Flanking residues** |
| Core | 39 | Y | IVGGVYLLP-R-RGPRLGVRA |
|  | 40 | Y | VGGVYLLPR-R-GPRLGVRAT |
|  | 43 | Y | VYLLPRRGP-R-LGVRATRKT |
|  | 47 | Y | PRRGPRLGV-R-ATRKTSERS |
|  | 50 | Y | GPRLGVRAT-R-KTSERSQPR |
|  | 55 | Y | VRATRKTSE-R-SQPRGRRQP |
|  | 59 | Y | RKTSERSQP-R-GRRQPIPKA |
|  | 61 | Y | TSERSQPRG-R-RQPIPKARR |
|  | 62 | Y | SERSQPRGR-R-QPIPKARRP |
|  | 69 | Y | GRRQPIPKA-R-RPEGRSWAQ |
|  | 70 | Y | RRQPIPKAR-R-PEGRSWAQP |
|  | 74 | Y | IPKARRPEG-R-SWAQPGYPW |
|  | 101 | Y | GWAGWLLSP-R-GSRPSWGPT |
|  | 104 | Y | GWLLSPRGS-R-PSWGPTDPR |
|  | 113 | Y | RPSWGPTDP-R-RRSRNLGKV |
|  | 114 | Y | PSWGPTDPR-R-RSRNLGKVI |
|  | 115 | Y | SWGPTDPRR-R-SRNLGKVID |
|  | 117 | Y | GPTDPRRRS-R-NLGKVIDTL |
|  | 149 | Y | VGAPLGGVA-R-ALAHGVRVL |
| NS3/4A | 24 | Y | IITSLTG-**R**-DKNQVEG |
|  | 62 | N | VYHGAGT-**R**-TIASPKG |
|  | 123 | N | VRRRGDS-**R**-GSLLSPR |
|  | 155 | Y | GHAVGIF-**R**-AAVCTRG |
|  | 462 | Y | AVSRTQR-**R**-GRTGRGK |
|  | 467 | Y | QRRGRTG-**R-**GKPGIYR |
|  | 474 | Y | RGKPGIY-**R**-FVAPGER |
|  | 512 | Y | TPAETTV-**R**-LRAYMNT |
|  | 514 | Y | AETTVRL-**R**-AYMNTPG |
|  | 570 | Y | YQATVCA-**R**-AQAPPPS |
|  | 587 | Y | QMWKCLI-**R**-LKPTLHG |
|  | 659 | Y | GCVVIVG-**R**-IVLSGKP |
|  | 677 | Y | PDREVLY-**R**-EFDEMEE |
| NS5A | 41 | N | IPFVSCQ-**R**-GYRGVWR |
|  | 44 | N | VSCQRGY-**R**-GVWRGDG |
|  | 56 | N | GDGIMHT-**R**-CHCGAEI |
|  | 160 | Y | LDGVRLH-**R**-FAPPCKP |
|  | 220 | Y | AAGRRLA-**R**-GSPPSVA |
|  | 304 | N | SVPAEIL-**R**-KSRRFAP |
|  | 356 | Y | SPPVPPP-**R**-KKRTVVL |
| NS5B | 109 | Y | GYGAKDV-**R**-CHARKAV |
|  | 154 | N | VQPEKGG-**R**-KPARLIV |
|  | 158 | Y | KGGRKPA-**R**-LIVFPDL |
|  | 168 | Y | VFPDLGV-**R**-VCEKMAL |
|  | 200 | Y | FQYSPGQ-**R**-VEFLVQA |
|  | 270 | N | GGPLTNS-**R**-GENCGYR |

Table S8:

Predicted ubiquitination sites in HCV C, NS3/4A, NS5A and NS5B. Conserved sites are highlighted in grey colour (Table S8).

| \|  \| Table S8 \|  \| \| --- \| --- \| --- \| \| Peptide (Core) \| **Position** \| **conservation** \| \| MSTNPKPQRKTKR \| 6 \| Y \| \| TNPKPQRKTKRNTNR \| 10 \| Y \| \| PKPQRKTKRNTNRRP \| 12 \| Y \| \| LGVRATRKTSERSQP \| 51 \| Y \| \| GRRQPIPKARRPEGR \| 67 \| Y \| \| Peptide (NS3/4A) \| Position \| conservation \| \| VYHGAGSKTLAGPKG \| 62 \| N \| \| SKTLAGPKGPVXQMY \| 68 \| N \| \| WPAPPGAKSLTPCTC \| 92 \| N \| \| PRPISTLKGSSGGPX \| 136 \| **Y** \| \| VCTRGVAKAVDFVPV \| 165 \| **Y** \| \| HAPTGSGKSTKVPAA \| 210 \| **Y** \| \| TGSGKSTKVPAAYAA \| 213 \| **Y** \| \| AYAAQGYKVLVLNPS \| 224 \| **N** \| \| GFGAYMSKAHGIDPN \| 244 \| **N** \| \| GEIPFYGKAIPLEXI \| 352 \| **N** \| \| AIPLEXIKGGRHLIF \| 360 \| **Y** \| \| HLIFCHSKKKCDELA \| 371 \| **Y** \| \| LIFCHSKKKCDELAA \| 372 \| **Y** \| \| IFCHSKKKCDELAAK \| 373 \| **Y** \| \| KCDELAAKLXXLGLN \| 380 \| **N** \| \| AHFLSQTKQAGENFX \| 551 \| **Y** \| \| WKCLIRLKPTLHGPT \| 589 \| **Y** \| \| GRIVLSGKPAVIPDR \| 665 \| **N** \| \| NS5A \|  \|  \| \| FKTWLKAKLLPXLPG \| 26 \| **Y** \| \| LSCQRGYKGVWRGDG \| 44 \| **N** \| \| PIPAPNYKRALWRVS \| 107 \| **N** \| \| HRFAPPCKPLLRXEV \| 166 \| **N** \| \| QLSAPSLKATCTTHH \| 240 \| **N** \| \| ECLRXXRKFPPALPI \| 308 \| **N** \| \| PPLLETWKXPDYXPP \| 330 \| **Y** \| \| PVPPPRRKRTVVLTE \| 358 \| **N** \| \| ALAELAEKSFGQSSE \| 378 \| **N** \| \| NS5B \|  \|  \| \| PCAAEEEKLPINPLS \| 20 \| **N** \| \| RSASQRQKKVTFDRL \| 50 \| **Y** \| \| SASQRQKKVTFDRLQ \| 51 \| **Y** \| \| XDVLKEXKARASXVK \| 72 \| **Y** \| \| KARASXVKARLLSVE \| 79 \| **N** \| \| PPHSARSKFGYGAKD \| 100 \| **Y** \| \| SKFGYGAKDVRSLSS \| 106 \| **Y** \| \| DVRSLSSKAXNHINS \| 114 \| **N** \| \| NHINSVWKDLLEDSX \| 124 \| **N** \| \| VFCVDPEKGGRKPAR \| 151 \| **Y** \| \| DPEKGGRKPARLIVY \| 155 \| **Y** \| \| LGVRVCEKRALYDXV \| 172 \| **Y** \| \| EFLLKAWKSKKTPMG \| 209 \| **N** \| \| LLKAWKSKKTPMGFS \| 211 \| **Y** \| \| LKAWKSKKTPMGFSY \| 212 \| **Y** \| \| DLDPEARKAIXSLTE \| 251 \| **N** \| \| GGPMXNSKGQXCGYR \| 270 \| **N** \| \| NTLTCYIKAXAACRA \| 298 \| **Y** \| \| VAHDASGKRVYYLTR \| 379 \| **N** \| \| RVAXCLRKLGVPPLR \| 491 \| **Y** \| \| RARAVRAKLIAQGGR \| 510 \| **N** \| \| GRAAICGKYLFNWAV \| 523 \| **N** \| \| WAVXTKLKLTPLPAA \| 535 \| **N** \|   **l** |
| --- | --- | --- | --- | --- | --- | --- | --- | --- | --- | --- | --- | --- | --- | --- | --- | --- | --- | --- | --- | --- | --- | --- | --- | --- | --- | --- | --- | --- | --- | --- | --- | --- | --- | --- | --- | --- | --- | --- | --- | --- | --- | --- | --- | --- | --- | --- | --- | --- | --- | --- | --- | --- | --- | --- | --- | --- | --- | --- | --- | --- | --- | --- | --- | --- | --- | --- | --- | --- | --- | --- | --- | --- | --- | --- | --- | --- | --- | --- | --- | --- | --- | --- | --- | --- | --- | --- | --- | --- | --- | --- | --- | --- | --- | --- | --- | --- | --- | --- | --- | --- | --- | --- | --- | --- | --- | --- | --- | --- | --- | --- | --- | --- | --- | --- | --- | --- | --- | --- | --- | --- | --- | --- | --- | --- | --- | --- | --- | --- | --- | --- | --- | --- | --- | --- | --- | --- | --- | --- | --- | --- | --- | --- | --- | --- | --- | --- | --- | --- | --- | --- | --- | --- | --- | --- | --- | --- | --- | --- | --- | --- | --- | --- | --- | --- | --- | --- | --- | --- | --- | --- | --- | --- | --- | --- | --- | --- | --- | --- | --- | --- |
|  |
